# HPV Status and Genotype Associations with Nodal Involvement and Treatment Strategy in Bulgarian Patients with Head and Neck Squamous Cell Carcinoma: A Prospective Multicenter Observational Study

**DOI:** 10.3390/medicina62061022

**Published:** 2026-05-25

**Authors:** Elitsa Deliverska, Vessela Raykova, Deyan Neichev, Stanislav Yordanov, Daniel Markov, Svetoslav Slavkov, Maria Aleksandrova, Viktor Lenkov, Zdravka Pashova-Tasseva

**Affiliations:** 1 Department of Dental, Oral and Maxillofacial Surgery, Faculty of Dental Medicine, Medical University of Sofia, 1000 Sofia, Bulgaria; elitsa.deliverska@fdm.mu-sofia.bg; 2 Department of Medical Microbiology, Medical Faculty, Medical University of Sofia, 1431 Sofia, Bulgaria; pumpi@abv.bg; 3 Department of Dental, Oral and Maxillofacial Surgery, Faculty of Dental Medicine, Medical University of Plovdiv, 4002 Plovdiv, Bulgaria; deyan.neychev@mu-plovdiv.bg; 4 Department of ENT Diseases, University Hospital St. Anna, 1709 Sofia, Bulgaria; stanio_hr@icloud.com; 5 Department of Maxillofacial Surgery, Pirogov Hospital, 1606 Sofia, Bulgaria; dvmarkov@yahoo.com (D.M.); slavkov1970@gmail.com (S.S.); 6 Faculty of Dental Medicine, Medical University of Sofia, 1431 Sofia, Bulgaria; mimi.aleksandrova.2006@gmail.com (M.A.); viktorlenkovv@gmail.com (V.L.); 7 Department of Periodontology, Faculty of Dental Medicine, Medical University of Sofia, 1000 Sofia, Bulgaria

**Keywords:** head and neck squamous cell carcinoma, human papillomavirus status, HPV genotype, nodal involvement, treatment strategy

## Abstract

*Background and Objectives*: Head and neck squamous cell carcinoma (HNSCC) is a biologically heterogeneous malignancy with variable clinical behavior and prognosis. Human papillomavirus (HPV)-associated tumors represent a distinct subgroup; however, data from Eastern European populations remain limited. This study aimed to evaluate the association between HPV DNA status and nodal involvement in a Bulgarian HNSCC cohort and to explore whether HPV genotype distribution is related to nodal involvement and therapeutic strategy. *Materials and Methods*: A prospective multicenter observational study with a cross-sectional analytical endpoint was conducted. Fifty patients with histologically confirmed HNSCC were included. Clinical and pathological data were collected, and HPV detection and genotyping were performed using molecular-based methods. Associations between HPV-related variables, nodal status (N0 vs. N+), and treatment strategy were evaluated using univariate tests. HPV status reflects DNA detection only and does not confirm transcriptionally active infection. *Results*: HPV DNA positivity was identified in 15/50 patients (30.0%). A higher proportion of nodal involvement was observed among HPV-positive patients compared with HPV-negative patients (46.7% vs. 17.1%, *p* = 0.040; crude OR = 4.23); however, this finding may be influenced by anatomical site distribution. In unadjusted analysis, HPV DNA positivity showed a relationship with nodal involvement (crude OR = 4.23; *p* = 0.040), although this should be interpreted cautiously. Multivariable analysis was not performed due to the limited number of outcome events. Differences in treatment allocation were observed between HPV-positive and HPV-negative patients; however, this finding may be confounded by anatomical site distribution and likely reflects differences in tumor localization rather than HPV-specific effects. Genotype analysis revealed heterogeneity, including multiple HPV types. *Conclusions*: HPV DNA positivity was observed in relation to nodal involvement in unadjusted analysis; however, this finding may be confounded by anatomical site and should be considered exploratory.

## 1. Introduction

Head and neck squamous cell carcinoma (HNSCC**)** represents a heterogeneous group of malignant diseases characterized by high incidence, unfavorable prognosis, and substantial disease-related mortality worldwide [1]. This heterogeneity is reflected in both clinical presentation and treatment response, as well as in the underlying molecular mechanisms driving tumor initiation and progression [2]. Among these factors, human papillomavirus (HPV**)**, particularly the high-risk genotype HPV16, has been established as a key etiological determinant in a distinct subset of HNSCC, most notably in oropharyngeal squamous cell carcinoma (OPSCC**)** [3]. HPV-associated tumors constitute a biologically distinct entity with unique molecular profiles and clinical behavior compared with HPV-negative disease [3].

The global prevalence of HPV-related OPSCC has increased substantially in recent decades, highlighting its growing clinical relevance [4,5]. At the molecular level, HPV-related carcinogenesis has been described in prior studies as involving viral oncoproteins such as E6 and E7; however, these mechanisms are presented here as contextual background and were not directly assessed in the present study [5,6]. Clinically, HPV-associated tumors are frequently characterized by early and often extensive cervical lymph node involvement, sometimes disproportionate to the size of the primary tumor [5,6]. This pattern has important implications for staging, prognosis, and treatment planning. In this context, nodal involvement (N+**)** remains one of the most important prognostic factors in HNSCC, being strongly associated with reduced survival and increased risk of recurrence [7]. While nodal involvement is a well-established prognostic factor in HNSCC, the present study is limited to cross-sectional assessment and does not evaluate survival or long-term outcomes. Beyond nodal status alone, additional parameters such as extranodal spread, lymph node yield, and lymph node ratio further reflect tumor aggressiveness and prognostic stratification [7,8]. Consequently, accurate assessment of nodal metastases is central to clinical decision-making, including surgical management and the indication for adjuvant therapy [9].

Beyond its etiological role, HPV status and genotype have been increasingly investigated as potential markers of tumor behavior and prognosis. While predictive models incorporating HPV-related variables have been developed in other HPV-associated malignancies, their applicability to HNSCC remains uncertain [10]. In addition, host–virus interactions may influence disease progression, as suggested by associations between HPV-related parameters and systemic inflammatory or immune-related indices [11]. In contrast, HPV-negative tumors are characterized by distinct molecular alterations unrelated to viral oncogenesis, contributing to heterogeneity in clinical outcomes [12,13].

Recent evidence suggests that integrating molecular and viral characteristics with conventional histopathological parameters may improve risk stratification in HNSCC [14]. In this context, HPV status and genotype are increasingly considered components of biology-driven models of tumor behavior rather than mere indicators of viral exposure [10,13]. Despite growing international evidence, data from Eastern European populations, including Bulgaria, remain limited. In particular, there is a lack of region-specific studies evaluating HPV prevalence, genotype distribution, and their potential association with nodal involvement and clinical management in HNSCC. Although HPV-related carcinogenesis is most strongly established in OPSCC, the present study was conducted in a broader HNSCC cohort, with anatomical site included as an analytical variable due to the limited number of OPSCC cases.

In light of these considerations, the aim of the present study was to evaluate the association between HPV DNA status and nodal involvement in a Bulgarian cohort of patients with HNSCC. A secondary objective was to explore whether HPV genotype distribution is descriptively associated with nodal involvement and therapeutic strategy. Given the limited number of HPV-positive cases, genotype-related analyses were considered exploratory.

## 2. Materials and Methods

### 2.1. Study Design

This was a prospective multicenter observational analytical study conducted between January 2024 and September 2025 at Alexandrovska Hospital, Pirogov Hospital and St. Anna Hospital clinic in Sofia. Patients were enrolled between January 2024 and September 2025. The study period commenced in January 2024 with a preparatory phase, including study design finalization, calibration procedures, and patient screening. Patient enrollment and data collection were initiated only after obtaining ethical approval (29 February 2024) and continued until September 2025. All eligible patients presenting during the recruitment period were consecutively enrolled to minimize selection bias. Recruitment was completed prior to data analysis. Given the limited number of outcome events, the study was not powered for inferential or predictive modeling, and all findings should be interpreted as exploratory. Due to the limited number of cases within individual anatomical subsites, the study was not powered for subsite-specific comparisons, and all analyses involving anatomical grouping should be interpreted cautiously.

This study represents a centralized analysis of individual data from patients diagnosed with head and neck squamous cell carcinoma (HNSCC) treated in three hospitals in Sofia, Bulgaria. A total of 50 cancer patients were enrolled. Eligible patients presenting during the recruitment period were invited to participate. Patients with HNSCC were stratified according to HPV status and genotype, and nodal involvement (N+ vs. N0) was defined as the primary outcome of interest. HPV testing was performed uniformly for all enrolled HNSCC patients as part of the study protocol, irrespective of tumor localization, stage, or clinical characteristics. For analytical purposes, anatomical site was included as a covariate to account for known differences in HPV prevalence and nodal behavior across subsites. This grouping was applied strictly for statistical adjustment and does not imply biological equivalence between oropharyngeal and laryngeal carcinomas. For analytical purposes, oropharyngeal tumors (OPSCC) were defined strictly based on anatomical localization within the oropharynx, while other pharyngeal and laryngeal tumors were analyzed as part of the upper aerodigestive tract group.

All participants agreed to participate in the study and signed an informed consent. The patients were recruited from Alexandrovska Hospital, Pirogov Hospital and St. Anna Hospital in Sofia.

### 2.2. Patients and Eligibility Criteria

Inclusion Criteria

Patients were eligible for inclusion if they met the following criteria:

- Age ≥ 18 years;
- Histologically confirmed head and neck squamous cell carcinoma (HNSCC);
- Tumor localization in the oral cavity, oropharynx or larynx;
- Availability of HPV testing results and clinical staging data, including TNM and nodal (N) status.

Exclusion Criteria

Patients were excluded if they met any of the following criteria:

- Prior oncological treatment (surgery, radiotherapy, or chemotherapy) before HPV sampling;
- Missing key clinical data, including HPV status or nodal (N) status.

### 2.3. Clinical and Histopathological Data

For each patient, clinical and pathological data were collected, including:

- Demographic characteristics (age and sex);
- Tumor localization, categorized as OPSCC or non-OPSCC;
- Tumor staging according to the TNM classification, according to the AJCC TNM classification, 7th edition, including T category, nodal (N) status, and overall stage;
- Histopathological grade of differentiation (G1–G3);
- Tumor growth pattern, classified as exophytic, infiltrative, or ulcero-infiltrative, according to histopathological assessment.

### 2.4. HPV Detection and Genotyping

HPV status was determined using molecular-based methods from clinical samples obtained according to a standardized protocol. In the present study, HPV status was defined based on PCR-based detection of viral DNA, which reflects viral presence but does not confirm transcriptionally active infection or HPV-related carcinogenesis. HPV testing was performed using oral rinse, brush biopsy, or combined sampling approaches; therefore, not all HPV-positive results were derived from tumor-specific material. HPV testing was performed uniformly for all enrolled HNSCC patients as part of the study protocol, irrespective of tumor localization or stage. To reduce the risk of false-negative results, particularly for oropharyngeal lesions, HPV testing was performed using oral rinse, brush biopsy, or a combined sampling approach, depending on lesion localization. In cases of suspected oropharyngeal tumors, sampling was preferentially performed using brush-based or lesion-directed methods rather than oral rinse alone. Furthermore, given the use of oral rinse and brush-based sampling methods, the possibility of detecting non-tumor-associated (bystander or passenger) HPV cannot be fully excluded.

HPV detection was performed using polymerase chain reaction (PCR)-based assays targeting conserved regions of the HPV genome. For HPV-positive samples, genotyping was subsequently carried out using a predefined panel of high-risk HPV genotypes. The specific PCR platform and target genes are described in detail in the study methodology. HPV status in the present study reflects DNA detection only and does not confirm transcriptionally active infection. The sensitivity and specificity of non-invasive HPV detection methods may vary compared to tissue-based assays and are generally considered lower for identifying tumor-specific HPV infection.

For the purpose of statistical analysis, patients were categorized according to HPV status as HPV-negative (HPV−) or HPV-positive (HPV+), based on PCR results. HPV-positive cases were further stratified into genotype groups as follows: HPV16-only, non-16 high-risk genotypes, and multiple genotype infections (coinfections), when applicable.

HPV genotyping and quantification were performed using multiplex real-time PCR with a validated commercial kit (HPV Quant 21, DNA-Technology, Moscow, Russia). The assay enables simultaneous quantitative detection and genotyping of 21 HPV types, including high-risk genotypes (16, 18, 26, 31, 33, 35, 39, 45, 51, 52, 53, 56, 58, 59, 66, 68, 73, 82) and low-risk genotypes (6, 11, 44). We acknowledge that established approaches for confirming transcriptionally active HPV infection include p16 immunohistochemistry and E6/E7 mRNA analysis. These methods were not performed in the present study due to the observational design and real-world clinical setting. Therefore, the results should be interpreted as reflecting HPV DNA positivity rather than confirmed HPV-related tumors.

DNA extraction was carried out using the PREP-NA/NA PLUS kit (DNA-Technology, Russia) according to the manufacturer’s protocol. Briefly, 100 μL of processed clinical material was subjected to lysis, internal control addition, incubation at 65 °C, precipitation, and sequential washing steps. Nucleic acids were eluted in dilution buffer and immediately used for PCR/real-time PCR analysis or stored at −20 °C until processing.

DNA was extracted using the PREP-NA/NA PLUS kit (DNA-Technology, Russia) following the manufacturer’s instructions. A 100 μL quantity of processed clinical material was mixed with lysis buffer and internal control, incubated at 65 °C, and subjected to precipitation and sequential washing. Nucleic acids were eluted in 50 μL (PREP-NA) or 300 μL (PREP-NA PLUS) of dilution buffer, vortexed, incubated, and centrifuged. The resulting DNA was immediately used for PCR/RT-PCR or stored at −20 °C (DNA up to 1 month; RNA up to 7 days) until analysis. HPV positivity was defined according to the manufacturer’s recommended thresholds for the applied assay, in accordance with standard laboratory practice. Amplification of the human β-globin gene was used as an internal control to verify DNA integrity, confirm adequate cellular content, and exclude false-negative results related to PCR inhibition or sample preparation errors. All molecular analyses were performed at the Department of Microbiology, Faculty of Medicine, Medical University of Sofia.

HPV positivity was defined as HPV DNA detection by multiplex real-time PCR (HPV Quant 21). HPV status in this study reflects viral DNA detection only and does not imply confirmation of transcriptionally active infection. p16 immunohistochemistry and E6/E7 mRNA assays were not performed in the present cohort. Therefore, HPV DNA positivity in this study may reflect tumor-associated or incidental oral HPV presence and should be interpreted accordingly. All PCRs were performed according to the manufacturer’s instructions, including specified cycling conditions and reagent concentrations as provided in the commercial kit protocols. Thermal cycling was carried out on a validated real-time PCR platform compatible with the HPV Quant 21 assay. Positive and negative controls were included in each run to ensure analytical reliability.

Accordingly, all HPV-related findings in this study are interpreted with caution and are not intended to establish causality or confirm HPV-related oncogenesis.

### 2.5. Treatment and Therapeutic Strategy

In the present analysis, the therapeutic strategy was defined according to the type and extent of the treatment actually administered. The primary representation of treatment approach was based on the type of surgical intervention performed, including excision, incision, hemilaryngectomy, or total laryngectomy.

For each patient, detailed treatment-related data were collected, including:

- Type and extent of surgical procedure;
- Management of the neck, including the performance of neck dissection (yes/no and type, when available);
- Administration of adjuvant therapy, including radiotherapy (RT) and/or chemoradiotherapy (CRT).

For the purpose of statistical analysis, treatment strategies were coded into three categories:

- Surgery alone;
- Surgery followed by adjuvant radiotherapy and/or chemoradiotherapy;
- Non-surgical or primary combined treatment, when applicable.

### 2.6. Study Endpoints

The primary endpoint of the study was the presence of nodal involvement, defined as nodal involvement (N+) versus absence of nodal disease (N0), reflecting tumor dissemination and disease aggressiveness.

Secondary endpoints included:

- Tumor stage according to the TNM classification;
- Histopathological characteristics, including grade of differentiation and tumor growth pattern;
- Therapeutic strategy, categorized according to predefined treatment groups, as a clinically relevant surrogate of disease severity and biological behavior.

No survival or recurrence outcomes were collected as part of the present cross-sectional analysis.

Given the limited number of OPSCC cases (n = 10), separate site-specific OPSCC-only analyses were not statistically feasible; therefore, tumor localization was included as an analytical covariate (upper aerodigestive tract vs. oral cavity) in regression modeling.

### 2.7. Statistical Analysis

Statistical analyses were performed using SPSS version 20.0 (IBM Corp., Armonk, NY, USA). Descriptive statistics were applied to summarize demographic, clinical, and pathological characteristics. Continuous variables were presented as mean ± standard deviation (SD) or median with interquartile range (IQR), depending on data distribution, while categorical variables were expressed as counts and percentages (n, %).

Associations between HPV-related variables (HPV status and genotype categories) and clinicopathological parameters were evaluated using univariate statistical tests, including the chi-square test or Fisher’s exact test for categorical variables and Student’s t-test or Mann–Whitney U test for continuous variables, as appropriate.

Given the limited number of outcome events, multivariable analysis was not performed, and all analyses were restricted to univariate exploratory methods. HPV status and genotype categories were included as independent variables. Results were reported as odds ratios (ORs) with corresponding 95% confidence intervals (95% CIs). A two-sided *p* value < 0.05 was considered statistically significant. Formal goodness-of-fit testing was not performed due to limited model stability. Association between categorical variables was evaluated using the chi-square test or Fisher’s exact test, as appropriate.

## 3. Results

### 3.1. Cohort Characteristics

A total of 50 patients with histologically confirmed head and neck squamous cell carcinoma (HNSCC) were included in the analysis. Among them, 10 patients (20.0%) presented with OPSCC, while 40 patients (80.0%) had tumors localized in the oral cavity. The mean age of the cohort was 61.97 ± 10.93 years. Male patients accounted for 80%, whereas female patients represented 20% of the study population.

**Figure 1:**
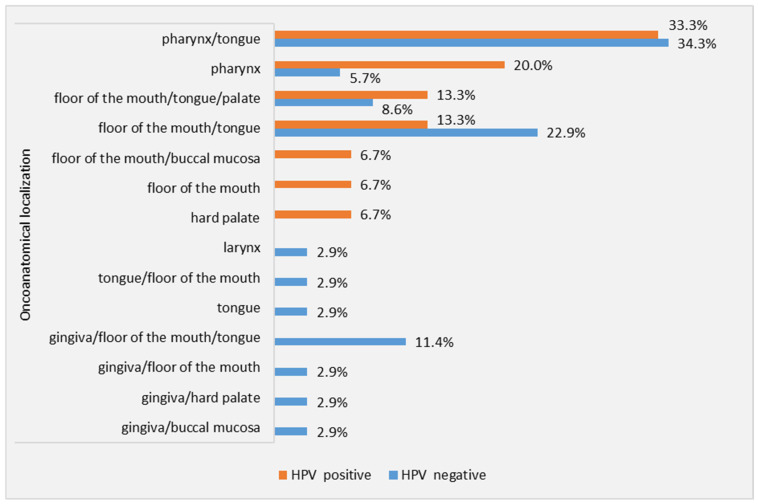
Oncoanatomical distribution of tumors according to HPV status.

**Figure A1:**
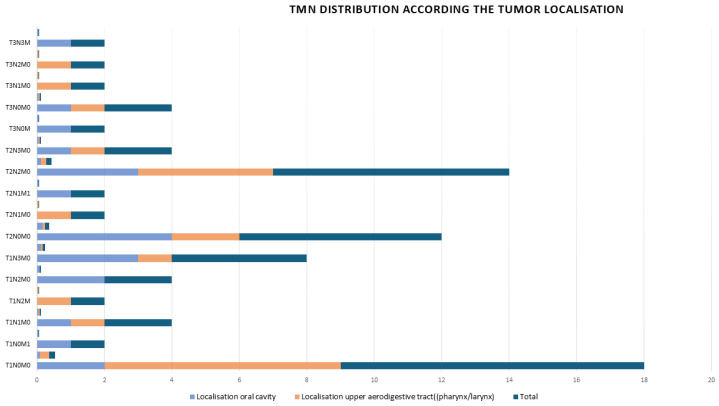
Distribution of TNM categories according to tumor localization (oral cavity vs. upper aerodigestive tract).

**Figure A2:**
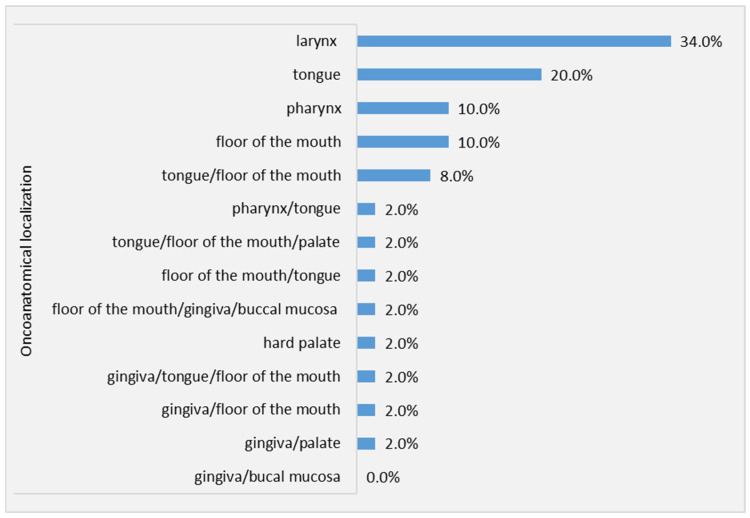
Oncoanatomical distribution of tumors in the study cohort.

HPV positivity was detected in 15 patients (30.0%), while 35 patients (70.0%) were classified as HPV-negative. Among the 10 patients diagnosed with OPSCC, 8 cases (80.0%) were HPV-positive, whereas only 2 (20.0%) were HPV-negative. Baseline clinical and histopathological characteristics stratified by HPV status are summarized in Table 1. For descriptive purposes, pharyngeal and laryngeal tumors were grouped within the upper aerodigestive tract category; this classification was applied for analytical adjustment only and does not imply biological equivalence between subsites. To further illustrate the anatomical distribution patterns according to HPV status, tumor localization was graphically stratified by HPV exposure (Figure 1). HPV-positive tumors were more frequently located in pharyngeal regions, whereas HPV-negative cases showed a broader distribution across oral cavity sites. This distribution supports the inclusion of tumor localization as an adjustment variable in Figure 1. Because OPSCC cases were limited, comparisons were performed in the overall cancer cohort, while anatomical site distribution by HPV status is presented to support subsequent adjustment. The TNM distribution and the overall oncoanatomical distribution of tumors in the cohort are shown in Appendix A (Figure A1) and Appendix B (Figure A2).

In Figure 1, the oncoanatomical distribution of tumors according to HPV status is presented. For descriptive purposes, tumors of the oropharynx, pharynx, and larynx were grouped within the upper aerodigestive tract category; this classification was applied for analytical adjustment only and does not imply biological equivalence between subsites. The borderline *p*-value for tumor localization (*p* = 0.050) should be interpreted with caution, given the small sample size and multiple comparisons.

### 3.2. HPV Genotype Profile in HPV-Positive Cases

Among HPV-positive patients, a heterogeneous distribution of HPV genotypes was observed. HPV16 represented the predominant genotype, accounting for the majority of HPV-positive cases, while non-16 high-risk genotypes were identified less frequently. In addition, a subset of patients harbored multiple HPV genotypes, indicating the presence of coinfections within the cohort.

The genotype distribution is summarized in Table 2. These descriptive data provide an overview of genotype heterogeneity within the cohort and serve as a contextual basis for exploratory analyses examining potential relationships with nodal involvement and therapeutic strategy. These observations are purely descriptive and, given the limited number of HPV-positive cases and genotype-specific detections, do not allow any meaningful genotype-stratified inference. Low-risk HPV genotypes (e.g., HPV6 and HPV11) were included as part of the detection panel; however, their presence was not interpreted as biologically equivalent to high-risk HPV types in relation to tumor behavior. The detection of low-risk genotypes, such as HPV6, reflects viral presence but does not imply a role in oncogenic processes.

The small number of detections for individual non-16 genotypes precludes any interpretation regarding their potential association with nodal involvement or treatment patterns. HPV16 was the predominant genotype and was detected across all anatomical subsites, whereas non-16 high-risk genotypes were observed less frequently and showed a more heterogeneous distribution. The distribution of HPV genotypes varied across anatomical subsites. Oropharyngeal tumors demonstrated the greatest genotype diversity, accounting for nearly half of all detected genotypes. HPV16 was the predominant genotype and was identified across all evaluated anatomical locations, including the oropharynx, oral cavity, and larynx. In contrast, non-16 high-risk genotypes were detected less frequently and showed a more restricted distribution across tumor sites. Detection of low-risk genotypes (e.g., HPV6) most likely represents incidental oral presence without oncogenic contribution in this cohort.

### 3.3. HPV Status and Nodal Involvement (Primary Endpoint)

Given the established prognostic relevance of nodal involvement in HNSCC, we next evaluated the association between HPV status and the presence of cervical lymph node metastases. The distribution of nodal status (N0 vs. N+) according to HPV status is summarized in Table 3.

A higher proportion of nodal involvement was observed among HPV-positive patients. However, this association should be interpreted with caution, as it may be substantially influenced by anatomical site distribution, particularly the predominance of HPV-positive tumors in oropharyngeal locations. Given the study design, it is not possible to distinguish whether this relationship reflects HPV-related biological effects, tumor location, or a combination of both. However, this association should be interpreted with caution, as it may be largely driven by anatomical site distribution, particularly the predominance of HPV-positive tumors in oropharyngeal locations.

When descriptively examined by tumor localization, the association between HPV status and nodal involvement appeared more pronounced in OPSCC cases; however, the small number of site-specific events precluded formal stratified statistical testing. Associations between categorical variables were evaluated using Fisher’s exact test due to small cell counts.

Building on the distribution of HPV genotypes among HPV-positive cases (Table 2) and the observed association between HPV status and nodal involvement. A higher proportion of nodal involvement was observed among HPV-positive patients in unadjusted analysis; however, this finding should be interpreted with caution, as it may be confounded by anatomical site distribution. Given the limited number of HPV-positive cases, genotype-related analyses in this study remain exploratory and descriptive.

Although the present dataset does not allow for definitive genotype-specific risk estimates, the combined interpretation of genotype-related observations given in Table 2 remains descriptive and hypothesis-generating. Given the limited number of HPV-positive cases and nodal events, these findings should be interpreted cautiously and are considered exploratory.

### 3.4. HPV Status and Therapeutic Strategy

Given the established differences in disease presentation between HPV-positive and HPV-negative HNSCC, we next examined whether HPV status was associated with variations in therapeutic strategy. The distribution of surgical approaches according to HPV status is presented in Table 4.

Differences in treatment patterns were observed between HPV-positive and HPV-negative patients. However, this finding should be interpreted with caution, as it is likely substantially influenced by anatomical site, tumor stage, and resectability rather than reflecting an independent effect of HPV status. No multivariable adjustment for anatomical site was performed for treatment allocation analysis due to limited sample size; therefore, site-related confounding cannot be excluded. Given the absence of adjustment for key clinical variables, including tumor localization and stage, no inference regarding the independent influence of HPV status on treatment decisions can be made.

## 4. Discussion

The present cohort provides descriptive data on HPV DNA positivity in HNSCC within a Bulgarian population, where approximately one-third of cases demonstrated HPV DNA detection. This observation is consistent with previous epidemiological studies reporting variable HPV prevalence and genotype diversity across European populations, with notable regional variability [15]. In the Bulgarian context, our findings are in line with recent national data indicating a measurable contribution of HPV to head and neck carcinogenesis [16].

HPV-related carcinogenesis is most clearly established in oropharyngeal squamous cell carcinoma (OPSCC), where transcriptionally active infection has been associated with distinct biological and clinical features. However, in the present study, HPV status was based on DNA detection only and does not confirm transcriptionally active or HPV-related tumors. Therefore, the findings should be interpreted as reflecting HPV DNA positivity rather than a biologically defined tumor subgroup.

Previous experimental and translational studies have described molecular and immunological differences between HPV-positive and HPV-negative tumors, including alterations in cell-cycle regulation, immune response, and tumor evolution [17]. In addition, advances in molecular targeting strategies have further emphasized the biological relevance of HPV in head and neck carcinogenesis [18]. These mechanisms are presented here for contextual background and were not directly assessed in the present study.

The present study design does not allow for the disentangling of the independent effects of HPV status and anatomical site. The observed association between HPV positivity and nodal involvement may therefore reflect the known concentration of HPV-positive tumors in oropharyngeal locations, which are inherently characterized by distinct patterns of nodal spread. Importantly, this observed association may be largely driven by anatomical site distribution, particularly the predominance of HPV-positive tumors in oropharyngeal locations, rather than reflecting an independent biological effect of HPV status. Not all HPV DNA-positive cases demonstrated nodal involvement in the present cohort, which may reflect biological and clinical heterogeneity. HPV positivity alone does not uniformly predict nodal dissemination and should be interpreted in conjunction with tumor- and host-related factors. These observations should be interpreted cautiously and do not imply a direct biologically driven mechanism, particularly given the use of HPV DNA detection in the present study. The following mechanistic considerations are presented for contextual background based on existing literature and were not directly assessed in the present study. From a mechanistic perspective, HPV DNA-positive carcinogenesis—particularly in tumors associated with the HPV16 genotype—exhibits distinct molecular features that have been proposed in prior studies as potential explanations for metastatic behavior. High-risk HPV16 exerts its oncogenic effects primarily through the E6 and E7 oncoproteins, leading to degradation of p53 and functional inactivation of the retinoblastoma pathway, with subsequent genomic instability and deregulated cell-cycle control [5,6]. In addition, HPV16 may indicate a higher propensity for viral genome integration into host DNA, a process linked to clonal selection, oncogene activation, and tumor progression, which may further contribute to aggressive regional dissemination [5]. Emerging evidence also indicates that HPV-associated tumors display unique interactions with the tumor immune microenvironment, including immune modulation and altered host–tumor signaling, potentially facilitating early lymphatic spread [6,19]. Because HPV-related disease is best characterized in OPSCC, we contextualize our findings primarily against OPSCC-focused literature, while acknowledging that our cohort includes additional head and neck sites. Clinical studies focusing on OPSCC have consistently reported a tendency toward early and extensive regional lymphatic dissemination in HPV-positive tumors, frequently disproportionate to the size and local extent of the primary lesion [20,21]. Meta-analytical data further support this observation, demonstrating higher rates of regional nodal involvement and distinct metastatic patterns in HPV-related disease, including involvement of retropharyngeal and distant nodal basins [22,23].

Importantly, this nodal behavior may reflect underlying biological mechanisms described in prior literature rather than purely anatomical factors. HPV DNA-positive tumors are characterized by distinct molecular and immunological features, including altered antigen presentation, immune cell infiltration, and cytokine signaling, which collectively shape tumor–host interactions and may facilitate early lymphatic spread [24]. These biological characteristics have been proposed as potential explanations for the clinical patterns reported in HPV-associated disease.

In this context, the present findings suggest a potential association between HPV DNA positivity and patterns of nodal involvement that may not be fully explained by tumor size alone in this cohort. While the advanced T category remains an established determinant of nodal dissemination, our results indicate that HPV DNA status may be associated with additional variability in nodal presentation. Given the limited number of outcome events, the present study was not powered for multivariable modeling; therefore, no independent effect of HPV status can be inferred. However, given that HPV status was based on DNA detection only, these findings should be interpreted with caution and do not imply a causal or biologically driven relationship.

In addition to the observed differences related to HPV status, analysis of the viral genotype spectrum in our cohort demonstrated pronounced heterogeneity among HPV-positive tumors, with a notable proportion of cases harboring multiple HPV genotypes. This finding indicates that HPV-associated HNSCC in our Bulgarian cohort cannot be regarded as a biologically uniform entity and highlights the clinical relevance of moving beyond a binary HPV-positive/negative classification. The detection of multiple genotypes suggests complex viral–host interactions that may influence tumor behavior, immune evasion, and metastatic potential.

Although prior studies suggest that specific high-risk HPV genotypes may be associated with distinct clinical phenotypes, the present dataset does not allow robust genotype-stratified statistical analysis. Given the small number of HPV-positive cases (n = 15), genotype-specific analyses are underpowered and should be considered hypothesis-generating at most. Therefore, genotype-related findings in this study should be interpreted as descriptive and hypothesis-generating rather than confirmatory. Large clinical studies have shown that HPV16-dominated tumors often display more homogeneous biological behavior, whereas non-16 high-risk genotypes and mixed infections define more heterogeneous and potentially less predictable disease courses [22,25]. In this context, the genotype heterogeneity observed in our cohort provides empirical support for the concept that HPV-positive HNSCC encompasses biologically diverse subgroups, even within relatively small and well-characterized patient populations.

Population-based investigations have further demonstrated marked geographic variability in oral HPV prevalence and genotype distribution, emphasizing that genotype-specific effects may differ across regions and populations [15]. The present findings therefore contribute region-specific data from Eastern Europe, a setting that remains underrepresented in the HPV–HNSCC literature. Such variability underscores the importance of local cohort analyses when interpreting the clinical relevance of HPV genotypes and cautions against direct extrapolation of genotype-related risk profiles from other populations.

The clinical interpretation of genotype heterogeneity is further complicated by reliance on surrogate biomarkers, particularly p16 immunohistochemistry, which may not uniformly reflect transcriptionally active, HPV-related oncogenesis across different populations [26]. Emerging molecular data suggest that genotype-specific differences in viral integration, oncogene expression, and early tumor evolution may shape metastatic behavior and disease progression [27]. Taken together, our findings support the added value of direct HPV genotyping, especially in cohorts where multiple genotypes and non-16 high-risk types are prevalent. Although genotype-specific associations in the present study remain exploratory, the observed heterogeneity reinforces the concept that genotype composition represents an additional layer of biological complexity with potential implications for refined risk stratification in HPV-associated HNSCC. Given the limited number of HPV-positive cases (n = 15), genotype-related observations should be interpreted as descriptive and hypothesis-generating.

Differences in therapeutic strategy observed in the present cohort further underscore the clinical relevance of HPV status beyond its biological and prognostic implications. In our analysis, HPV-positive and HPV-negative tumors were managed using distinct surgical approaches, reflecting differences in tumor presentation, nodal involvement, and clinician risk assessment. Differences in treatment allocation were observed between HPV-positive and HPV-negative patients; however, these differences likely reflect variations in anatomical distribution and clinical presentation rather than an independent effect of HPV status. In the absence of adjustment for key determinants such as tumor site, stage, and surgical feasibility, the present data do not support any conclusion regarding HPV status as an independent determinant of treatment strategy.

This observation is consistent with contemporary evidence demonstrating that HPV-associated OPSCC represents a distinct clinical entity with implications for treatment planning and outcome prediction [28,29]. Systematic reviews and meta-analyses have shown that HPV DNA-positive tumors, particularly those stratified by viral subtype, exhibit differential clinical outcomes, reinforcing the rationale for treatment individualization based on HPV-related parameters [28]. At the same time, the management of HPV-positive disease—especially in the presence of cervical lymph node metastasis—poses specific challenges, including the interpretation of p16 positivity, the influence of tobacco exposure, and the selection of appropriate candidates for treatment de-escalation [19].

Although de-escalation strategies are being actively investigated in HPV-associated OPSCC, the present study was not designed to evaluate treatment outcomes; therefore, no conclusions regarding therapeutic intensity or response can be drawn. However, the successful implementation of such approaches depends critically on accurate and standardized HPV detection and classification [30,31]. Variability in diagnostic algorithms, reliance on surrogate biomarkers, and population-specific differences in HPV biology may contribute to heterogeneity in treatment decisions across centers and regions. In this context, emerging molecular and diagnostic advances—including improved viral detection methods, integration profiling, and epigenetic biomarkers—are expected to refine patient stratification and support more precise therapeutic planning [27,32].

Taken together, the association between HPV status and therapeutic strategy observed in the present cohort aligns with an evolving paradigm in head and neck oncology, in which biological markers increasingly complement traditional TNM-based frameworks. Our findings suggest that HPV-related stratification may be associated with differences in clinical management patterns; however, these differences likely reflect anatomical distribution and real-world practice rather than a direct biological determination by HPV status. Future prospective studies integrating standardized viral testing, genotype profiling, and molecular biomarkers will be essential to determine how HPV status and genotype composition can be systematically incorporated into treatment algorithms, particularly in the context of personalized and de-escalated therapeutic strategies.

Several limitations of the present study should be acknowledged. First, the cohort size was relatively modest and derived from a single population, which may limit the generalizability of the findings. Second, although comprehensive clinical and pathological data were available, certain subgroup analyses—particularly those involving HPV genotype stratification—were exploratory in nature and should be interpreted with caution. In addition, treatment-related analyses reflected real-world clinical practice rather than standardized protocol-driven interventions, limiting conclusions regarding treatment efficacy. Importantly, anatomical grouping in the present study was applied for statistical purposes only and should not be interpreted as reflecting biological equivalence between oropharyngeal and laryngeal carcinomas.

HPV status was defined based on PCR-based detection of HPV DNA, which identifies viral presence but does not establish biologically active HPV DNA-positive oncogenesis. Therefore, the results should be interpreted as reflecting HPV DNA positivity rather than definitively confirmed HPV DNA-positive tumors.

HPV detection was based on DNA PCR without confirmation of transcriptionally active infection, which limits the biological interpretation of HPV DNA-positive disease. Given the exploratory nature of the adjusted analyses and absence of survival data, these findings should be interpreted as hypothesis-generating rather than definitive. The relatively small sample size and low number of outcome events limit statistical robustness and preclude definitive conclusions, particularly for genotype-specific and treatment-related analyses. No clinical recommendations can be derived from the present findings.

Despite these limitations, the study has notable strengths. The cohort was well characterized with detailed clinical, pathological, and HPV-related data, allowing integrated analysis of HPV status, genotype heterogeneity, nodal involvement, and therapeutic strategy. Importantly, the study provides population-specific data from Bulgaria, a region that remains underrepresented in the HPV–HNSCC literature. The simultaneous evaluation of biological markers and real-world treatment patterns represents an additional strength, offering clinically relevant insights that bridge molecular characterization and practical decision-making.

## 5. Conclusions

In conclusion, HPV DNA positivity was associated with nodal involvement in unadjusted analysis; however, this finding may be confounded by anatomical site distribution and does not support inference of an independent association. Given the limited number of outcome events and the absence of multivariable modeling, the results should be interpreted cautiously and considered exploratory. Genotype profiling revealed heterogeneity among HPV-positive tumors; however, genotype-related findings remain descriptive and hypothesis-generating. The present study was not designed to evaluate survival or treatment response, and no conclusions regarding prognosis or therapeutic implications can be drawn. Overall, the findings provide region-specific data on HPV DNA positivity in HNSCC and suggest that both HPV status and genotype composition may contribute to variability in clinical presentation. Further studies with larger, well-characterized cohorts and standardized HPV assessment are required to clarify these relationships. These findings are exploratory and hypothesis-generating. Our findings based on HPV DNA detection cannot distinguish causal infection from incidental oral HPV presence. Therefore, they should not be used to infer HPV-driven oncogenesis. The present findings may serve to generate hypotheses for future prospective studies rather than inform clinical decision-making.

## Figures and Tables

**Table 1:** Baseline Clinical and Tumor Characteristics of the Study Cohort According to HPV Status.

Characteristic	Total (N = 50)	HPV− (n = 35)	HPV+ (n = 15)	*p*-Value
Age, years (mean ± SD)	—	61.97 ± 10.93	—	0.684
Sex, n (%)				0.436
Male	40 (80.0)	30 (83.3)	10 (71.4)	
Female	10 (20.0)	6 (16.7)	4 (28.6)	
Tumor localization, n (%)				0.050
Upper aerodigestive tract (including oropharynx, pharynx, and larynx)	10 (20.0)	2 (5.7)	8 (53.3)	
Oral cavity	40 (80.0)	33 (94.3)	7 (46.7)	
N status, n (%)				0.040
N0	37 (74.0)	29 (82.9)	8 (53.3)	
N+	13 (26.0)	6 (17.1)	7 (46.7)	
Histological grade, n (%)				0.215
G1–G2	40 (80.0)	31 (86.1)	9 (64.3)	
G3	10 (20.0)	5 (13.9)	5 (35.7)	
Growth pattern, n (%)				0.704
Exophytic or infiltrative	10 (20.0)	8 (22.2)	2 (14.3)	
Ulcero-infiltrative	40 (80.0)	28 (77.8)	12 (85.7)	

Abbreviations: HPV, human papillomavirus; OPSCC, oropharyngeal squamous cell carcinoma; SD, standard deviation; NS, not significant. Values are presented as n (%) unless otherwise indicated. Comparisons were performed using the chi-square test or Fisher’s exact test, as appropriate. Anatomical grouping was applied for analytical purposes only and does not imply biological equivalence between subsites.

**Table 2:** Distribution of HPV Genotype Detection Counts in HPV-Positive Cases according to anatomical site, tumor stage, and nodal status in HPV-positive tumors.

HPV Genotype	Genotype Detections (n)	Oropharyngeal (OPSCC)	Oral Cavity (OSCC)	Larynx	T1–T2	T3–T4	N0	N+
HPV16	12	5	2	5	8	4	2	10
HPV6	3	2	0	1	3	0	1	2
HPV39	2	0	2	0	1	1	0	2
HPV58	2	0	2	0	1	1	0	2
HPV52	1	1	0	0	0	1	0	1
HPV31	1	1	0	0	1	0	0	1
HPV66	1	1	0	0	0	1	1	0
HPV51	1	1	0	0	0	1	1	0
Total genotype detections	23	11	6	6	14	9	5	11

Because several tumors harbored multiple HPV genotypes, the total number of genotype detections exceeds the number of HPV-positive patients (n = 15). Values are presented as n (%) unless otherwise indicated. Comparisons were performed using the chi-square test or Fisher’s exact test, as appropriate.

**Table 3:** Association between HPV Status and Nodal involvement (N+).

HPV Status	N0, n (%)	N+, n (%)	Total (n)	Crude OR (95% CI)	*p*-Value
HPV−	29 (82.9)	6 (17.1)	35	Reference	
HPV+	8 (53.3)	7 (46.7)	15	4.23 (1.10–16.25)	0.040
Total	37 (74.0)	13 (26.0)	50	—	—

Abbreviations: HPV, human papillomavirus; OR, odds ratio; CI, confidence interval. Values are presented as n (%) unless otherwise indicated. Comparisons were performed using the chi-square test or Fisher’s exact test, as appropriate. The reported odds ratio reflects an unadjusted analysis and should be interpreted cautiously. The wide confidence interval reflects the limited sample size and should be interpreted with caution.

**Table 4:** Clinical Management Approach According to HPV Status.

Therapeutic Approach	HPV− (n = 35)	HPV+ (n = 15)	Total (N = 50)	*p*-Value
Excision/resection in clear margins	7 (20.0%)	8 (53.3%)	15 (30.0%)	
Diagnostic procedure (incisional biopsy)	14 (40.0%)	3 (20.0%)	17 (34.0%)	
Hemilaryngectomy	8 (22.9%)	1 (6.7%)	9 (18.0%)	
Total laryngectomy	6 (17.1%)	3 (20.0%)	9 (18.0%)	
Overall comparison	—	—	—	0.045

Abbreviations: HPV, human papillomavirus. Values are presented as n (%) unless otherwise indicated. Comparisons were performed using the chi-square test or Fisher’s exact test, as appropriate.

## Data Availability

The raw data supporting the conclusions of this article will be made available by the authors upon reasonable request.

## Abbreviations

The following abbreviations are used in this manuscript:

HPV	Human Papillomavirus
HNSCC	Head and Neck Squamous Cell Carcinoma
OPSCC	Oropharyngeal Squamous Cell Carcinoma
OR	Odds Ratio
CI	Confidence Interval

## Appendix A

The figure illustrates differences in TNM patterns between tumors arising in the oral cavity and those of the upper aerodigestive tract (pharynx and larynx).

## Appendix B

The figure presents the overall distribution of tumor localizations within the oral cavity and upper aerodigestive tract in the entire cohort.
